# Adeno-Associated Virus Serotype 8-Mediated Genetic Labeling of Cholangiocytes in the Neonatal Murine Liver

**DOI:** 10.3390/pharmaceutics12040351

**Published:** 2020-04-13

**Authors:** Sanghoon Lee, Ping Zhou, Senyo Whyte, Soona Shin

**Affiliations:** 1 Division of Pediatric General and Thoracic Surgery, Cincinnati Children’s Hospital Medical Center, Cincinnati, OH 45229, USA; 2 Department of Surgery, University of Cincinnati College of Medicine, Cincinnati, OH 45229, USA; 3 Medical Scientist Training Program, University of Cincinnati College of Medicine, Cincinnati, OH 45229, USA; 4 Molecular and Developmental Biology Graduate Program, Cincinnati Children’s Hospital Medical Center, Cincinnati, OH 45229, USA

**Keywords:** adeno-associated virus, neonate, cholangiocyte, liver, transduction, viral vector

## Abstract

Determination of the cellular tropism of viral vectors is imperative for designing precise gene therapy. It has been widely accepted that transduction of hepatocytes using adeno-associated virus serotype 8 (AAV8) is a promising approach to correct inborn errors in neonates, but the type of neonatal hepatic cells transduced by AAV8 has not been thoroughly investigated. To address this question, we used a reporter mouse that carries Cre recombinase (Cre)-inducible yellow fluorescent protein (YFP). Our analysis primarily focused on cholangiocytes, given their pivotal roles in normal liver function and disease. We treated *Rosa^YFP/+^* mice at postnatal day 2 (P2) with AAV8-cytomegalovirus (CMV) promoter-Cre and analyzed livers at P10 and P56. The vast majority of HNF4α+ hepatocytes were labeled with YFP at both time points, and 11.6% and 24.4% of CK19+ cholangiocytes were marked at P10 and P56, respectively. We also detected YFP+ cells devoid of hepatocyte and cholangiocyte markers, and a subset of these cells expressed the endothelial and fibroblast marker CD34. Next, we used the hepatocyte-specific thyroxine-binding globulin (TBG) promoter. Surprisingly, AAV8-TBG-Cre marked 6.8% and 30.9% of cholangiocytes at P10 and P56, respectively. These results suggest that AAV8 can be a useful tool for targeting cholangiocytes in neonatal livers.

## 1. Introduction

Adeno-associated virus vectors serotype 8 (AAV8) has been widely used to transduce hepatocytes [1,2,3,4,5,6], the main parenchymal cells in the liver. Due to its liver tropism, low immunogenicity, low toxicity, and high transduction efficiency of hepatocytes, AAV8 has been considered an ideal method to target hepatocytes [7,8]. Neonatal injections of AAV, associated with attenuated neutralizing antibody responses, have been proposed as a promising strategy to correct inborn diseases with early onset [9,10,11,12,13].

Previous studies have demonstrated that the transduction efficiency and tropism of AAV vectors depend on several variables [8,14,15]. These include capsid serotypes, promoters, vector dosage, routes of injection, and the timing of injection, suggesting the importance of characterizing responses to AAV in the neonatal liver. It is becoming increasingly clear that non-hepatocytes in the liver such as cholangiocytes, endothelial cells, and fibroblasts contribute to normal liver function as well as disease progression [16,17,18,19,20], implying that non-specific infections of non-parenchymal cells may lead to side effects. Conversely, if AAV8 transduces non-parenchymal cells, this may lead to a broader application of AAV8 vectors. Therefore, a precise understanding of the cellular tropism of AAV8 can allow for improved study design and gene therapy. However, whether AAV8 has the capability to target non-hepatocytes at neonatal ages remains unclear.

Therefore, we hypothesized that AAV8 can be used for transducing non-hepatocytes in the neonatal liver and aimed to characterize the cellular tropism of AAV8 using Cre recombinase driven by the cytomegalovirus (CMV) and thyroxine-binding globulin (TBG) promoters [21]. The CMV promoter leads to ubiquitous expression, while the latter has been used to target hepatocytes [6,21]. Given the critical role of biliary epithelia in liver disease and normal bile flow [17,20], this study aimed to determine hepatic cell types transduced by AAV8 following neonatal injections with a focus on cholangiocytes (biliary epithelial cells). Our study reveals a novel use of AAV8 (i.e., a tool for the genetic labeling of cholangiocytes in the neonatal murine liver). 

## 2. Materials and Methods 

### 2.1. Animal Experiment

*Rosa^YFP/YFP^* mice (The Jackson Laboratory, Bar Harbor, ME) were crossed with C57BL/6J (The Jackson Laboratory, Bar Harbor, ME) mice to generate *Rosa^YFP/+^* mice [22]. AAV8-CMV-red fluorescent protein (RFP), AAV8-CMV-Cre, AAV8-TBG-LacZ (encodes β-galactosidase), and AAV8-TBG-Cre viral preps were produced by Addgene (Watertown, MA; Addgene viral prep numbers: 105548-AAV8, 105537-AAV8, 105534-AAV8, and 107787-AAV8, respectively) using plasmids gifted by Dr. James M. Wilson to Addgene. AAV8 vectors were diluted in saline to a total volume of 50uL. *Rosa^YFP/+^* mice were given intraperitoneal injections of 2.0 × 10^11^ genome copies at P2 with the day of birth defined as P0 [23,24]. Tissues were harvested 8 days and 54 days after injection, at P10 and P56, respectively. Both male and female neonates were included in the experiment: (a) AAV8-CMV-RFP: 6 males and 4 females were analyzed at P10; (b) AAV8-CMV-Cre: 3 males and 8 females were analyzed at P10, and 2 males and 3 females were analyzed at P56; (c) AAV8-TBG-LacZ: 3 males and 4 females were analyzed at P10; (d) AAV8-TBG-Cre: 2 males and 6 females were analyzed at P10, and 6 males and 1 female were analyzed at P56. The protocol was approved by the Institutional Animal Care and Use Committee of the Cincinnati Children’s Hospital Medical Center (IACUC2018-0074, approved 9 November 2018).

### 2.2. Immunofluorescence 

Paraffin-embedded formalin-fixed sections were dewaxed and rehydrated sections were subjected to antigen retrieval followed by incubation in blocking solution (3% normal donkey serum and 0.25% triton X-100 in phosphate-buffered saline) for 1 hour at room temperature. Next, sections were treated with primary antibodies (Table 1) for overnight at 4 °C and secondary antibodies for 2 h at room temperature. 4′,6-diamidino-2-phenylindole (DAPI) was used for nuclei staining. 

### 2.3. Quantification and Data Analysis

In P10 livers the average numbers of cells analyzed per animal were 4448 ± 443 hepatocyte nuclear factor 4 alpha (HNF4α)+ hepatocytes and 398 ± 105 cytokeratin 19 (CK19)+ cholangiocytes (mean ± SD). In P56 livers the average numbers of cells analyzed per animal were 14,859 ± 2421 HNF4α+ hepatocytes and 1076 ± 246 CK19+ cholangiocytes (mean ± SD). The percentage of YFP-marked cells within the HNF4α+ and CK19+ populations were calculated, and all data were expressed as mean ± SD. Image J (National Institutes of Health, Bethesda, Maryland) was used for cell counting [25]. Prism 8 (GraphPad Software, Inc., San Diego, CA, USA) was used for Student’s *t*-test and Mann-Whitney U test. A p-value of smaller than 0.5 was considered significant in this study. 

## 3. Results

### 3.1. Neonatal Injection of AAV8-CMV-Cre Labels Cholangiocytes

**Figure 1:**
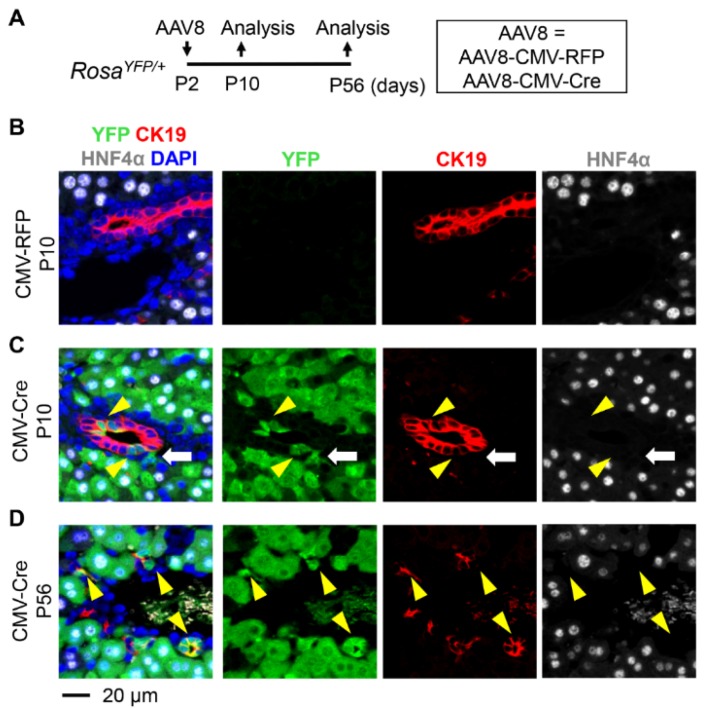
Labeling of cholangiocytes by injection of AAV8-CMV-Cre at P2. (**A**) Schematic representation of the treatment and analysis protocol. *Rosa^YFP/+^* reporter mice were injected with AAV8-CMV-Cre at postnatal day 2 (P2) and tissues were analyzed at P10 and P56, respectively. (**B**–**D**) Immunostaining analysis. No YFP-labeled cells were detected in the liver of *Rosa^YFP/+^* mice treated with the control vector AAV8-CMV-RFP (**B**). AAV8-CMV-Cre labeled CK19-expressing cholangiocytes and HNF4α-expressing hepatocytes (**C**,**D**). Yellow arrowheads: YFP+CK19+ cholangiocytes. White arrow: YFP+ cells that do not express CK19 and HNF4α. 4′,6-diamidino-2-phenylindole (DAPI) was used for nuclei staining.

**Figure 2:**
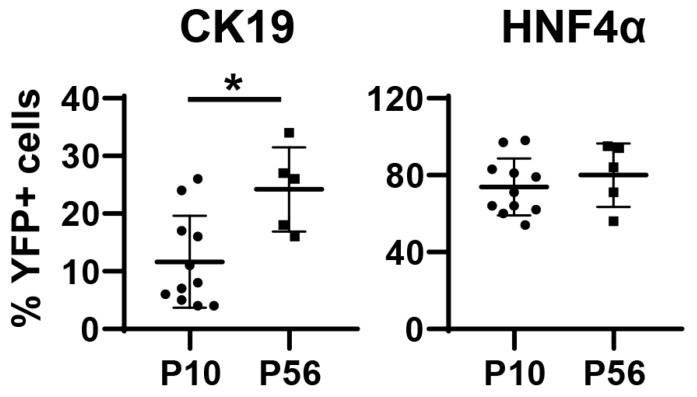
Quantification of the percentage of AAV8-CMV-Cre/YFP-labeled cells within CK19-expressing cholangiocytes and HNF4α-expressing hepatocytes. Error bars represent the standard deviation of the mean (*n* = 5–11 mice per group). * *p* < 0.05.

To determine the transduction specificity of AAV8 in neonatal livers, we used *Rosa^YFP/+^* reporter mice [22]. Although the *Rosa26* locus is ubiquitously expressed, transcriptional stop sequences block expression of yellow fluorescent protein (YFP). Removal of floxed stop sequences by Cre recombinase leads to permanent labeling of transduced cells with YFP. Therefore, we treated postnatal day 2 (P2) *Rosa^YFP/+^* mice with AAV8-CMV-Cre and livers were analyzed at P10 (Figure 1A). To determine the efficiency of transduction, we performed immunostaining for YFP, hepatocyte marker HNF4α, and cholangiocyte marker CK19. HNF4α and CK19 expressions were mutually exclusive, and the majority of hepatocytes were labeled as expected, while the control vector AAV8-CMV-RFP did not lead to YFP expression (Figure 1B,C). Surprisingly, we also detected Cre/YFP-marked cholangiocytes at P10 with 11.6% ± 7.8% (mean ± SD) labeling efficiency (Figure 1C and Figure 2). 

**Figure 3:**
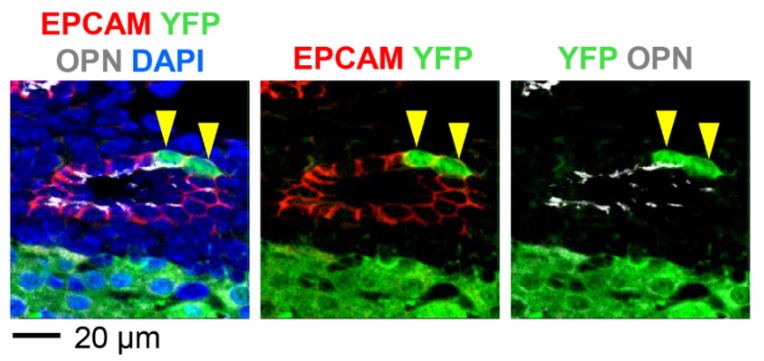
AAV8-CMV-Cre labels cholangiocytes expressing EPCAM and OPN. *Rosa^YFP/+^* reporter mice were injected with AAV8-CMV-Cre at P2 and livers were analyzed at P10. OPN marks the apical surface of cholangiocytes. Yellow arrowheads: YFP+EPCAM+OPN+ cells.

To determine whether cholangiocytes remain labeled at a later time point, we treated *Rosa^YFP/+^* animals with AAV8-CMV-Cre at P2 and analyzed the liver at P56 (Figure 1A). While most hepatocytes were labeled at this time point, 24.4% ± 7.5% of CK19+ cells were also labeled (Figure 1D and Figure 2). Interestingly, there was a statistically significant increase in the percentage of labeled cholangiocytes from P10 to P56 (Figure 2). YFP-labeled cholangiocytes also expressed additional markers for cholangiocytes, epithelial cell adhesion molecule (EPCAM) and osteopontin (OPN) (Figure 3) [26,27,28]. Our results indicate that neonatal injection of AAV8 can be used to transduce a substantial fraction of cholangiocytes.

### 3.2. AAV8-CMV-Cre Labels CD34-Expressing Cells

**Figure 4:**
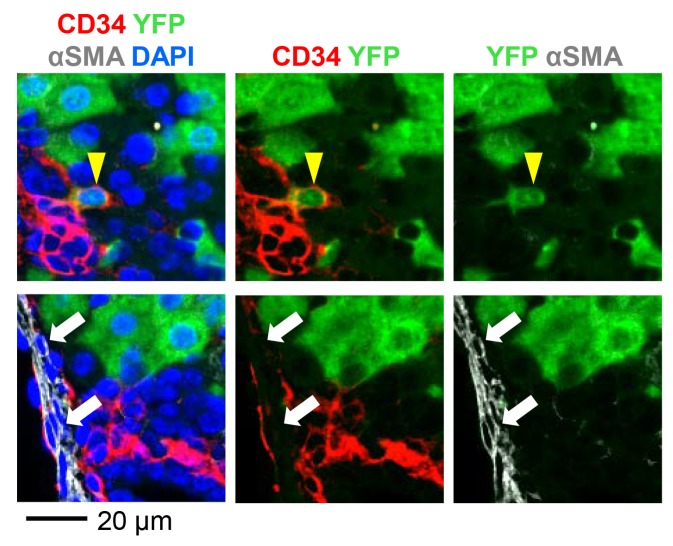
*Rosa^YFP/+^* reporter mice were injected with AAV8-CMV-Cre at P2 and livers were analyzed at P10. Yellow arrowhead: YFP+CD34+αSMA- cell (upper panel). White arrows: YFP-CD34-αSMA+ cells (lower panel).

We observed that a subset of YFP-marked cells expressed neither HNF4α nor CK19 (Figure 1C, white arrow). Therefore, we performed immunostaining of P10 livers for additional markers (Figure 4). Livers were stained for the fibroblast marker alpha smooth muscle actin (αSMA) and CD34 that is expressed by the endothelium of central and portal veins as well as portal fibroblasts [16,29]. Although rare, we confirmed the presence of CD34+αSMA- cells labeled with YFP. On the other hand, none of the CD34-αSMA+ cells were marked, suggesting that AAV8 transduces distinct subsets of cells. This result indicates that AAV8-CMV-Cre labels CD34-expressing cells in addition to cholangiocytes. 

### 3.3. Neonatal Injection of AAV8-TBG-Cre Labels Cholangiocytes

**Figure 5:**
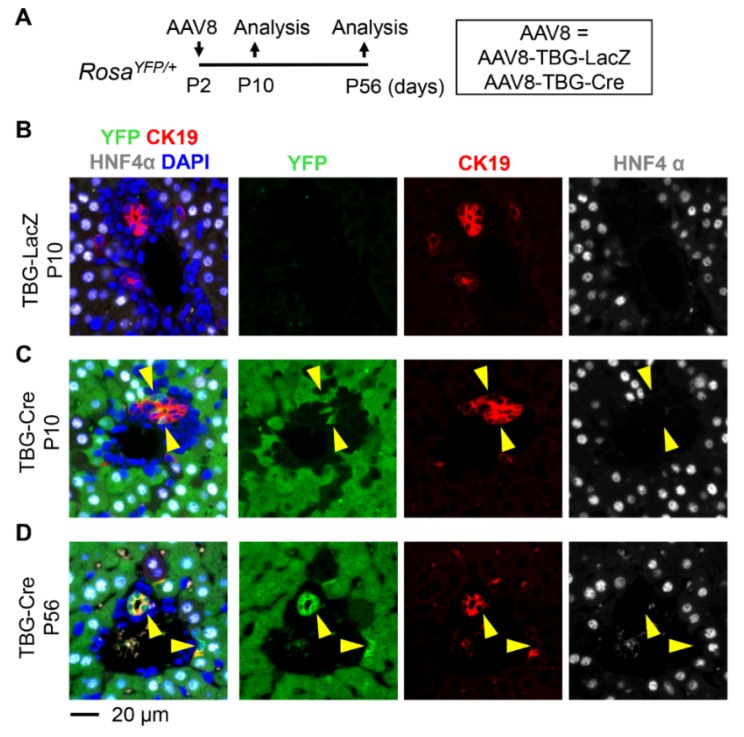
(**A**) Schematic representation of the treatment and analysis protocol. *Rosa^YFP/+^* reporter mice were injected with AAV8-TBG-Cre at P2 and tissues were analyzed at P10 and P56, respectively. (**B**–**D**) Immunostaining analysis. No YFP-labeled cells were detected in the liver of *Rosa^YFP/+^* mice treated with the control vector AAV8-TBG-LacZ (**B**). AAV8-TBG-Cre labeled CK19-expressing cholangiocytes and HNF4α-expressing hepatocytes (**C**,**D**). Yellow arrowheads: YFP+CK19+ cholangiocytes.

**Figure 6:**
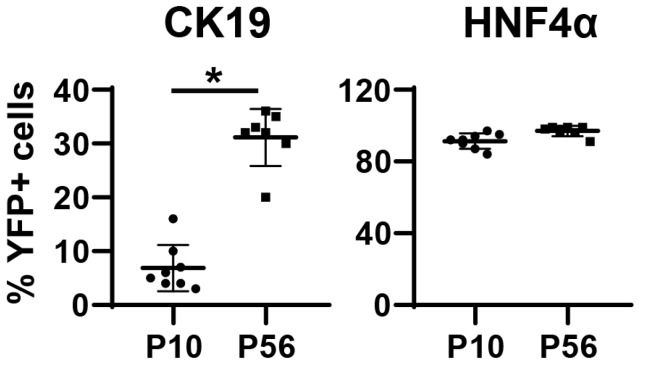
Quantification of the percentage of AAV8-TBG-Cre/YFP-labeled cells within CK19-expressing cholangiocytes and HNF4α-expressing hepatocytes. Error bars represent the standard deviation of the mean (*n* = 7–8 mice per group). * *p* < 0.05.

We then hypothesized that a hepatocyte-specific promoter would suppress the labeling of cholangiocytes by AAV8. To test this hypothesis, we used the TBG promoter to drive the expression of Cre (Figure 5A). When *Rosa^YFP/+^* mice were treated with AAV8-TBG-Cre at P2, the vast majority of hepatocytes were labeled, while the control vector AAV8-TBG-LacZ did not lead to the expression of YFP (Figure 5B–D). Surprisingly, 6.8% ± 4.2% and 30.9% ± 5.2% of CK19+ cholangiocytes were labeled at P10 and P56, respectively (Figure 5C,D and Figure 6). The percentage of labeled cholangiocytes increased from P10 to P56 in line with the previous pattern observed with the CMV promoter (Figure 2). The percentage of AAV8-TBG-Cre-marked CK19+ cells at P56 was slightly higher than the percentage of CK19+ cells marked by AAV8-CMV-Cre although the difference was not statistically significant (TBG: 30.9% ± 5.2% and CMV: 24.4% ± 7.5%, *p* = 0.08). This phenomenon might be attributable to the silencing of the CMV promoter [9]. 

In conclusion, our data indicate AAV8-CMV-Cre and AAV8-TBG-Cre can be used for the genetic labeling of cholangiocytes in neonatal murine livers. 

## 4. Discussion

This study investigated the cellular tropism of the AAV8 vector in the neonatal murine liver and revealed that (a) non-hepatocytes such as CK19+ cells and CD34+ cells can be transduced by AAV8 and (b) the TBG promoter can be used to label cholangiocytes. Both AAV8-CMV-Cre and AAV8-TBG-Cre marked CK19+ cholangiocytes with YFP when injected into *Rosa^YFP/+^* reporter mice at P2, with elevated labeling efficiency at P56 as compared to P10. One possible explanation of this increased percentage of labeled cholangiocytes is that AAV8-expressed Cre continues to mark CK19+ cholangiocytes after P10. Indeed, studies suggest that although AAV is primarily episomal, the expression of the transgene can last up to several months [15,30]. The association between the stability of transgenes and transduction efficiency requires further investigation.

To interpret outcomes of gene therapy and avoid adverse effects, it is critical to understand the cellular tropism of vectors in the neonatal liver. Our data not only provide insight into AAV-based therapy but also can be applied for fast genetic modulation of non-hepatocytes since the generation of genetically engineered mouse models can be time-consuming and expensive. However, our injection protocol has some limitations. First, neonatal injection of AAV is a suitable strategy for Cre-mediated permanent modification of the genome but not ideal for transient overexpression since the viral genome will be diluted as the liver expands [11]. Also, YFP-marked CD34+ cells were rare with a labeling efficiency of less than 1% (data not shown). Our protocol is not suitable for the modulation of the entire CD34+ population but may be useful for tracing the fate of individual CD34+ cells. 

Nevertheless, this study has established a novel strategy for labeling non-hepatocytes in neonatal livers. Our data also revealed a need for reevaluating the specificity of hepatocyte promoters, especially when the vector is administered at young ages. For therapeutic application of our transduction strategy, future research should investigate the use of cholangiocyte-specific promoters and species dependence of cellular tropism.

## 5. Conclusions

This study demonstrates that the AAV8 vector is a valuable tool for transducing cholangiocytes in vivo, and that the CMV and TBG promoters can be used to drive Cre expression in cholangiocytes. Our findings potentially provide a basis for fast and simple genetic engineering of cholangiocytes. 

## Figures and Tables

**Table 1:** Antibodies used for immunofluorescence.

Target	Host Species	Source (Catalog Number)	Dilution
YFP	Goat	Abcam (ab6673)	250
YFP	Chicken	Aves Labs (GFP-1020)	500
CK19	Rabbit	Abcam (ab52625)	200
EPCAM	Rabbit	Abcam (ab71916)	100
OPN	Goat	R&D Systems (AF808)	200
HNF4α	Mouse	R&D Systems (PP-H1415-00)	400
CD34	Rabbit	Abcam (ab81289)	100
αSMA	Mouse	Abcam (ab7817)	200
